# Research on Valveless Piezoelectric Pump Based on Coriolis Effect

**DOI:** 10.3390/mi16050527

**Published:** 2025-04-29

**Authors:** Qiufeng Yan, Zhiling Liu, Wanting Sun, Mengyao Jiang

**Affiliations:** 1 School of Electrical Engineering and Automation, Nantong University, Nantong 226019, China; yanqf@nuaa.edu.cn; 2 School Key Laboratory of Mechanics and Control for Aerospace Structures, Nanjing University of Aeronautics and Astronautics, Nanjing 210016, China; 3 Graduate School of Education, Joongbu University, Goyang 10279, Republic of Korea; liuzl044926@163.com; 4 School of Engineering, Lancaster University, Lancaster LA1 4YW, UK; sunwt_hit@126.com

**Keywords:** Coriolis force, arc-shaped tube, valveless piezoelectric pump (VLPP)

## Abstract

In previous studies, a valveless piezoelectric pump with arc-shaped tubes (VPPAST) based on the Coriolis Effect was proposed. To promote the application of VPPAST in the field of navigation and guidance, it is vital to further explore the influences of the layout and structural parameters of arc-shaped tubes on the flow rate. Accordingly, in this study, the analysis of flow characteristics of fluid in arc-shaped tubes was conducted, and the velocity difference between the clockwise and counterclockwise flow of the liquid was reduced. Eventually, the flow equations of three layout modes of arc-shaped tubes were established. VPPAST with anomalous-direction arc-shaped tubes, single-arc-shaped tube, and same-direction arc-shaped tubes were produced using 3D printing technology. In addition, the valveless piezoelectric pump with the anomalous-direction arc-shaped tubes (VLPPADA) with different parameter flow tubes were also fabricated. Based on the resultant flow rates of each piezoelectric pump, it was demonstrated that the flow rate of the VLPPADA was the highest under the same driving conditions, and the flow rate can be determined as 1.72 mL/min when the driving voltage was set as 160 V at 14 Hz. It indicated that the pump flow rate of VLPPADA was directly proportional to the base radius and width of the arc-shaped tube.

## 1. Introduction

Due to the advantages of a compact structure, low energy consumption, and high stability [1,2,3], the piezoelectric pump has found widespread application in the fields of biomedical science [4,5,6], microfluidics [7,8,9], and liquid cooling [10,11,12]. In terms of structural characteristics, piezoelectric pumps can be divided into valve piezoelectric pumps (VPPs) [13] and VLPPs [14]. VPPs have the weakness of “pump-lagging of valve” due to the removable valve body [15], which can affect the output performance of piezoelectric pumps. In contrast, VLPPs have attracted more attention from scholars.

Stemme et al. [16] designed a VLPP with diffuser/nozzle tubes, ushering the research on piezoelectric pumps into the valveless era. The maximum flow rate was 16 mL/min. Since then, extensive studies on the principle, structure, and performance of VLPPs with diffuser/nozzle tubes [2,17,18] have been carried out. Over the past few years, with the continuous presentation of the advantages of VLPPs, the VLPPs have aroused great concern. Jiang et al. [19] designed VLPPs with tesla tubes. Zhang et al. [20] developed a VLPP with Y-shape tubes. Subsequently, a VLPP with multistage Y-shape treelike bifurcate tubes was applied in the chip cooling [21,22], and the maximum flow rate was 35.6 mL/min. Zhang et al. [23,24] proposed a VLPP with an unsymmetrical slope chamber bottom, and the flow resistance structure inside the pump was integrated, leading to a more compact structure of the pump. Additionally, Bian et al. [25,26] designed a VLPP with streamline flow tubes, where the vortices in the flow tube could be reduced, and a peak flow rate of 142.0 mL/min was achieved when the voltage was 100 V_p-p_ at 80 kHz.

Coriolis found that in the Northern Hemisphere, the Coriolis force generated by the Earth’s rotation has an inhibitory effect on clockwise moving fluids and a promoting effect on counterclockwise moving fluids [27]. In addition, it was reported that there was secondary flow in the cross-section of a curved tube, which combined with the axial mainstream to form a spiral forward motion, resulting in improved fluid transport and mixing functions [28].

Based on the Coriolis Effect, we proposed a VPPAST [29,30], and a theoretical analysis and experimental study were conducted on the VPPAST. However, our previous research only conducted preliminary explorations. To further promote the application of the VPPAST, this manuscript focuses on studying the effects of structural parameters such as the flow tube layout, width, and base circle radius on flow rate.

In this work, we design the VLPP with anomalous-direction arc-shaped tubes, single-arc-shaped tubes, and same-direction arc-shaped tubes. The flow characteristics of fluid in the arc-shaped tubes are analyzed, and the velocity difference between the clockwise and counterclockwise flow of liquid can be reduced. In addition, the operating principle of the VPPAST is revealed, and the flow equations of three layout modes of arc-shaped tubes are established. The VLPPADA, the valveless piezoelectric pump with the single-arc-shaped tubes (VLPPSA), and the valveless piezoelectric pump with the same-direction arc-shaped tubes (VLPPSDA) were fabricated using the 3D printing technique, and their pump flow rates were compared in experiments. Eventually, the VLPPADA with different parameters was designed, and the influences of the base circle radius and channel width on the flow rate are explored through the experimental results.

## 2. Internal Flow Analysis Flow Tube

**Figure 1:**
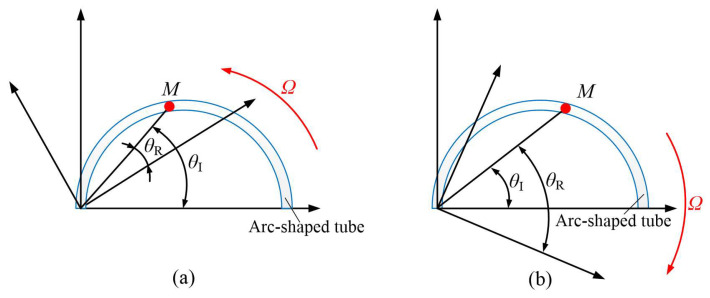
The diagrams of flow in the arc tube: (**a**) coordinate system rotates counter clockwise, (**b**) coordinate system rotates clockwise.

As shown in Figure 1, to analyze the flow characteristics of the liquid in the arc-shaped tube, a cylindrical coordinate system and a control equation were established. The research object of the geometric model is the arc curve of the plane. The curve equation of the arc is expressed as *R* = 2*acosθ*, where *R* is the radius of the planar circular arc curve, *a* is the radius of the base circle, and *θ* is the angle of the planar arc-shaped tube [29].

During the analysis, it is assumed that the liquid in the arc-shaped tube is under barotropic, uniform, incompressible, non-viscous ideal fluid. Based on the fundamental equations of fluid mechanics and the geometric characteristics of the arc-shaped tube, the differential equation of fluid in the coordinate system (*r*, *θ*, *z*) can be expressed as Equation (1) [29]:

(1) $$\left\{\begin{array}{l} \frac{du_{I}}{dt_{I}}-\frac{v_{I}^{2}}{r_{I}}=-\frac{\partial P_{I}}{\partial r_{I}}\\ \frac{dv_{I}}{dt_{I}}+\frac{u_{I}v_{I}}{r_{I}}=-\frac{1}{r_{I}}\frac{\partial P_{I}}{\partial \theta_{I}}\\ \frac{dw_{I}}{dt_{I}}=-\frac{\partial P_{I}}{\partial z_{I}} \end{array}\right.$$

where *I* is the quantity in the inertial frame of reference. *u*, *v*, and *w* are the velocity components of the three coordinate axes of the column coordinates, which are expressed as *r, θ,* and *z*, respectively [29]. Then,

(2) $$P_{I}=\frac{P}{\rho }+gz$$

where *ρ* is the density of the liquid in the flow tube, and *P* is the function of *ρ*.

In order to simplify, the liquid inside the flow tube is treated as a micro-unit in this study. If the position of the micro-unit remains unchanged, it can be assumed that the original cylindrical coordinate system is rotated, and thereby a new cylindrical coordinate system that rotates about the z axis at an angular velocity of *Ω* is formed. As shown in Figure 1a, when the cylindrical coordinate system is counterclockwise, the relationship between the rotating coordinate system (*r_R_*, *θ_R_*, *z_R_*) and the original coordinate system (*r_I_*, *θ_I_*, *z_I_*) can be expressed as Equation (3) [29]:

(3) $$\left\{\begin{array}{l} r_{R}=r_{I}=r\\ \theta_{R}=\theta_{I}-\Omega t_{I}\\ z_{R}=z_{I}=z\\ t_{R}=t_{I}=t \end{array}\right.$$

When *t* = 0, the rotating coordinate system is consistent with the original coordinate system. The velocity components on the coordinate axis of rotation (*r_R_*, *θ_R_*, *z_R_*) can be expressed as Equation (4) [29]:

(4) $$\left\{\begin{array}{l} u_{R}=\frac{dr_{R}}{dt_{R}}=\frac{dr_{I}}{dt_{I}}=u_{I}=u\\ v_{R}=\frac{d(r_{R}\theta_{R})}{dt_{R}}=\frac{d(r_{I}\theta_{I}-r_{I}\Omega t_{I})}{dt_{I}}=v_{I}-\Omega t\\ w_{R}=\frac{dw_{R}}{dt_{R}}=\frac{dw_{I}}{dt_{I}}=w_{I}=w \end{array}\right.$$

Substitute Equations (3) and (4) into Equation (1) and *dr*/*dt* = *u*. Thus, the motion equation of the fluid in the rotating coordinate system can be obtained as follows [29]:

(5) $$\left\{\begin{array}{l} \frac{du_{R}}{dt_{R}}-\frac{v_{R}^{2}}{r_{R}}-2\Omega v_{R}-\Omega^{2}r_{R}=-\frac{\partial P_{I}}{\partial r_{R}}\\ \frac{dv_{R}}{dt_{R}}+\frac{u_{R}v_{R}}{r_{R}}+2\Omega u_{R}=-\frac{1}{r}\frac{\partial P_{I}}{\partial \theta_{R}}\\ \frac{dw_{R}}{dt_{R}}=-\frac{\partial P_{I}}{\partial z_{R}} \end{array}\right.$$

Since *du_R_*_/_*dt_R_* = 0, *v* is represented without a subscript. Utilizing the first expression in Equation (5), we can express the equation for *v* as follows [29]:

(6) $$v^{2}+2\Omega rv+\Omega^{2}r^{2}=\frac{\partial P_{I}}{\partial t}\frac{\partial t}{\partial r}$$

Since the rate makes sense, *v*_1_ can be expressed as follows [29]:

(7) $$v_{1}=-2a\Omega \cos(\theta )+\sqrt{\frac{\partial P_{I}}{\partial t}\frac{\partial t}{\partial r}}$$

Similarly, as shown in Figure 1b, when the coordinate system rotates clockwise, the relation between the rotated coordinate system (*r_R_*, *θ_R_*, *z_R_*) and the original coordinate system (*r_I_*, *θ_I_*, *z_I_*) is expressed as follows [29]:

(8) $$\left\{\begin{array}{l} r_{R}=r_{I}=r\\ \theta_{R}=\theta_{I}+\Omega t_{I}\\ z_{R}=z_{I}=z\\ t_{R}=t_{I}=t \end{array}\right.$$

The derivation of the equation for *v* is as follows [29]:

(9) $$v^{2}-2\Omega rv+\Omega^{2}r^{2}=\frac{\partial P_{I}}{\partial t}\frac{\partial t}{\partial r}$$

Accordingly, *v*_2_ can be expressed as follows [29]:

(10) $$v_{2}=2a\Omega \cos(\theta )+\sqrt{\frac{\partial P_{I}}{\partial t}\frac{\partial t}{\partial r}}$$

In subtracting Equation (10) from Equation (7), Δ*v* can be calculated using Equation (11) [29]:

(11) $$\Delta v=v_{2}-v_{1}=4a\Omega \cos(\theta )$$

In summary, because of the presence of the Coriolis term, the velocity difference can be obtained between the clockwise and counter-clockwise flow of the liquid in the arc-shaped tube, and the velocity difference is determined as 4*aΩ*cos(*θ*).

## 3. Structure Design and Working Principle of the Pump

### 3.1. Structure Design

**Figure 2:**
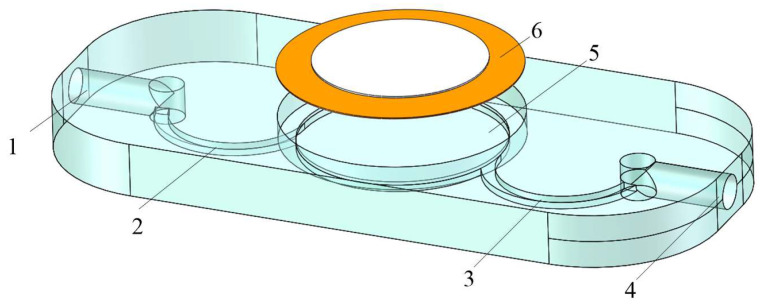
Structural diagram of VPPAST. 1. Inlet 2. Arc-shaped tubes of inlet. 3. Arc-shaped tubes of outlet. 4. Outlet. 5. Pump chamber. 6. PZT vibrator.

Figure 2 shows the structural diagram of the VPPAST. Such a piezoelectric pump is composed of a piezoelectric (PZT) vibrator and a pump body. Meanwhile, the pump body is made up of an inlet, an outlet, a pump chamber, inlet arc-shaped tubes, and outlet arc-shaped tubes. When the AC voltage is applied, the periodic vibration of the PZT actuator can cause changes in the volume of the pump chamber. Under the action of Coriolis force, the arc-shaped tube acts as a one-way valve, thereby serving as a “pump” within the entire structure.

### 3.2. Working Principle

The Coriolis force exerts an inhibition effect on clockwise-moving liquids and a promoting effect on counterclockwise-moving liquids. This results in different flow resistances when the liquid flows clockwise and counterclockwise in a circular flow tube. Under the periodic reciprocating vibration of the PZT vibrator, the liquid can generate unidirectional flow, and then the function of a pump is achieved. In order to analyze the influence of the layout of flow pipes on the flow rate of piezoelectric pumps on both sides of the pump chamber, the working principles of three types of the VPPAST with different layouts are analyzed, and the corresponding pump flow calculation formulas are established.

Based on the rotation velocity of the cylindrical coordinate system, the flow velocity of the liquid flowing clockwise and counterclockwise in the arc-shaped tube can be obtained by Equations (12) and (13), respectively:

(12) $$v_{c}=-2a\Omega \cos(\theta )+\sqrt{\frac{\partial P_{I}}{\partial t}\frac{\partial t}{\partial r}}$$

(13) $$v_{a}=2a\Omega \cos(\theta )+\sqrt{\frac{\partial P_{I}}{\partial t}\frac{\partial t}{\partial r}}$$

where *a* is the base circle radius, *Ω* is the angular velocity of liquid flow, *v_c_* is the velocity of liquid flow clockwise, and *v_a_* is the velocity of liquid flow counterclockwise.

In the suction struck, the flow rate of liquid flowing clockwise through the arc-shaped tube into the pump chamber is expressed by Equation (14):

(14) $$Q_{inc}=S\int \left[-2a\Omega \cos(\theta )+\sqrt{\frac{\partial P_{I}}{\partial t}\frac{\partial t}{\partial r}}\right]dt$$

where *S* is the cross-sectional area of the flow channel, and *t* is the time of liquid flow.

The flow rate of liquid flowing counterclockwise into the pump chamber through the arc-shaped tube is expressed by Equation (15).

(15) $$Q_{ina}=S\int \left[2a\Omega \cos(\theta )+\sqrt{\frac{\partial P_{I}}{\partial t}\frac{\partial t}{\partial r}}\right]dt$$

In the exhaust struck, the flow rate of liquid flowing clockwise through the arc-shaped tube out of the pump chamber is expressed by Equation (14).

(16) $$Q_{outc}=S\int \left[-2a\Omega \cos(\theta )+\sqrt{\frac{\partial P_{I}}{\partial t}\frac{\partial t}{\partial r}}\right]dt$$

The flow rate of liquid flowing counterclockwise through the arc-shaped tube out of the pump chamber can be expressed by Equation (17):

(17) $$Q_{outa}=S\int \left[2a\Omega \cos(\theta )+\sqrt{\frac{\partial P_{I}}{\partial t}\frac{\partial t}{\partial r}}\right]dt$$

**Figure 3:**
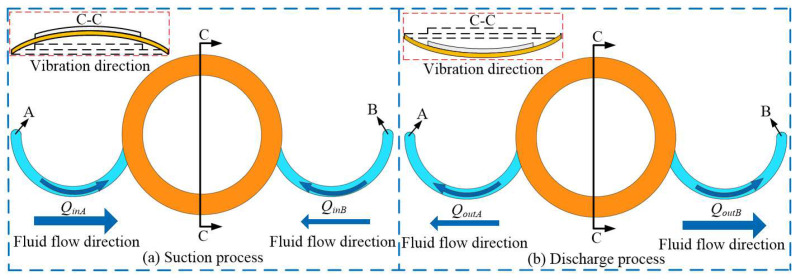
Working process of the VLPPADA.

Figure 3 shows the working process of VLPPADA. As shown in Figure 3a, the pump chamber is in the suction stage, and the piezoelectric vibrator vibrates upward. It should be noted that the volume of the pump chamber is increased, while the pressure is decreased. The liquid flows into the pump chamber from tube A and B, respectively. At this point, the liquid flows counterclockwise in tube A, and the Coriolis force plays a promoting role. Meanwhile, it flows clockwise in tube B, and the Coriolis force acts as a hindrance. The flow rates of liquid from tubes A and B into the pump chamber can be calculated by Equations (18) and (19), respectively:

(18) $$Q_{inA(H)}=S\int \left[2a\Omega \cos(\theta )+\sqrt{\frac{\partial P_{I}}{\partial t}\frac{\partial t}{\partial r}}\right]dt$$

(19) $$Q_{inB(H)}=S\int \left[-2a\Omega \cos(\theta )+\sqrt{\frac{\partial P_{I}}{\partial t}\frac{\partial t}{\partial r}}\right]dt$$

In Figure 3b, during the discharge process of the pump chamber, the piezoelectric vibrator vibrates downward, while the volume of the pump chamber decreases and the pressure increases. The liquid flows out of the pump chamber from tubes A and B, respectively. The liquid flows clockwise in tube A, and the Coriolis force plays an obstructive role. Meanwhile, it flows counterclockwise in tube B, and the Coriolis force plays a promoting role in this progress. The flow rates of liquid from tubes A and B out of the pump chamber can be expressed by Equations (20) and (21), respectively:

(20) $$Q_{outA(H)}=S\int \left[-2a\Omega \cos(\theta )+\sqrt{\frac{\partial P_{I}}{\partial t}\frac{\partial t}{\partial r}}\right]dt$$

(21) $$Q_{outB(H)}=S\int \left[2a\Omega \cos(\theta )+\sqrt{\frac{\partial P_{I}}{\partial t}\frac{\partial t}{\partial r}}\right]dt$$

In summary, the flow rate generated by the VLPPADA in one cycle is obtained by Equation (22).

(22) $$\begin{array}{l} Q_{H}=\left(Q_{inA(H)}-Q_{outA(H)}\right)-\left(Q_{inB(H)}-Q_{outB(H)}\right)\\ \begin{array}{c} \begin{array}{l} \;=S\int 8a\Omega \cos(\theta )dt\\ \;=8aS\sin(\Omega t) \end{array} \end{array} \end{array}$$

**Figure 4:**
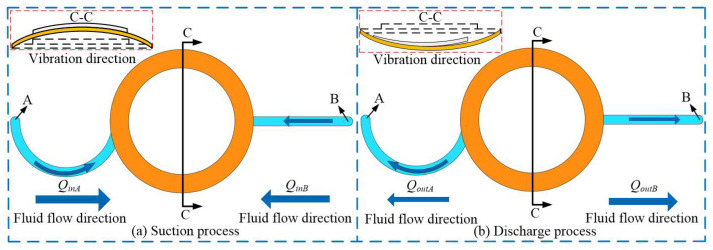
Working process of the VLPPSA.

Figure 4 shows the working process of VLPPSA. In Figure 4a, the pump chamber is in the suction stage, and the piezoelectric vibrator vibrates upward. It is found that the volume of the pump chamber is increased, and the pressure is decreased. The liquid flows into the pump chamber from tubes A and B, respectively. The liquid flows counterclockwise in flow tube A, and the Coriolis force plays a promoting role. In addition, the liquid flows in a straight line in flow tube B. The flow rates of liquid from tubes A and B into the pump chamber are calculated by Equations (23) and (24), respectively:

(23) $$Q_{inA(U)}=S\int \left[2a\Omega \cos(\theta )+\sqrt{\frac{\partial P_{I}}{\partial t}\frac{\partial t}{\partial r}}\right]dt$$

(24) $$Q_{inB(U)}=Sv_{t(in)}t$$

where *v_t_*_(in)_ is the velocity of the liquid flows in flow tube B.

In Figure 4b, the pump chamber is in the discharge process, and the piezoelectric vibrator vibrates downward. This demonstrates that the volume of the pump chamber is decreased, and the pressure is increased. The liquid flows out of the pump chamber from tubes A and B, respectively. The liquid flows clockwise in tube A, and the Coriolis force plays an obstructive role. In addition, the liquid flows in a straight line in flow tube B. The flow rates of liquid from tubes A and B out of the pump chamber are expressed by Equations (25) and (26), respectively:

(25) $$Q_{outA(U)}=S\int \left[-2a\Omega \cos(\theta )+\sqrt{\frac{\partial P_{I}}{\partial t}\frac{\partial t}{\partial r}}\right]dt$$

(26) $$Q_{outB(U)}=Sv_{t(out)}t$$

where *v_t_*_(out)_ is the velocity of the liquid flows in tube B.

Because tube B is a straight channel with an equal cross-section, the liquid velocity in the suction and discharge sections of tube B is equal; thus *v_t_*_(in)_ = *v_t_*_(out)_.

In summary, the flow rate generated by the VLPPSA in one cycle is expressed by the following Equation (27):

(27) $$\begin{array}{l} Q_{U}=\left(Q_{inA(U)}-Q_{outA(U)}\right)-\left(Q_{inB(U)}-Q_{outB(U)}\right)\\ \begin{array}{c} \begin{array}{l} \;=S\int 4a\Omega \cos(\theta )dt-S\left(v_{t(in)}-v_{t(out)}\right)\\ \;=4aS\sin(\Omega t) \end{array} \end{array} \end{array}$$

**Figure 5:**
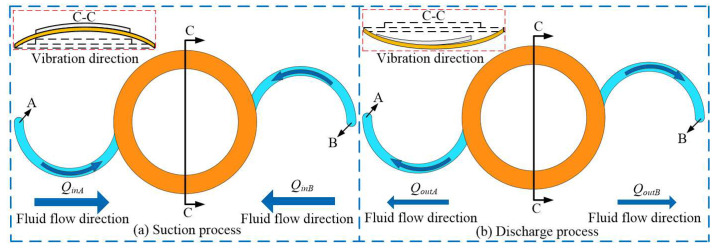
Working process of the VLPPSDA.

Figure 5 shows the working process of VLPPSDA. As shown in Figure 5a, the pump chamber is in the suction stage, and the piezoelectric vibrator vibrates upward. Meanwhile, the volume of the pump chamber is increased, and the pressure is decreased. The liquid flows into the pump chamber from tubes A and B, respectively. The liquid flows counterclockwise in tubes A and B, and the Coriolis force plays a promoting role. The flow rates of liquid from tubes A and B into the pump chamber can be calculated by Equations (28) and (29), respectively:

(28) $$Q_{inA(S)}=S\int \left[2a\Omega \cos(\theta )+\sqrt{\frac{\partial P_{I}}{\partial t}\frac{\partial t}{\partial r}}\right]dt$$

(29) $$Q_{inB(S)}=S\int \left[2a\Omega \cos(\theta )+\sqrt{\frac{\partial P_{I}}{\partial t}\frac{\partial t}{\partial r}}\right]dt$$

In Figure 5b, the pump chamber is in the discharge process, and the piezoelectric vibrator vibrates downward. This indicates that the volume of the pump chamber decreases, and the pressure increases. The liquid flows out of the pump chamber from tubea A and B, respectively. The liquid flows clockwise in flow tubes A and B, and the Coriolis force plays an obstructive role. The flow rates of liquid from tubes A and B out of the pump chamber are calculated by Equations (30) and (31), respectively:

(30) $$Q_{outA(S)}=S\int \left[-2a\Omega \cos(\theta )+\sqrt{\frac{\partial P_{I}}{\partial t}\frac{\partial t}{\partial r}}\right]dt$$

(31) $$Q_{outB(S)}=S\int \left[-2a\Omega \cos(\theta )+\sqrt{\frac{\partial P_{I}}{\partial t}\frac{\partial t}{\partial r}}\right]dt$$

In summary, the flow rate generated by the VLPPSDA in one cycle is obtained by Equation (32).

(32) $$\begin{array}{l} Q_{U}=\left(Q_{inA(U)}-Q_{outA(U)}\right)-\left(Q_{inB(U)}-Q_{outB(U)}\right)\\ \begin{array}{c} \begin{array}{l} \;=S\int 4a\Omega \cos(\theta )dt-S\int 4a\Omega \cos(\theta )dt\\ \;=0 \end{array} \end{array} \end{array}$$

Based on the above theoretical analysis, it can be concluded that the pump flow rates of the VLPPADA, the VLPPSA, and the VLPPSDA are determined as 8*aS*sin(*Ωt*), 4*aS*sin(*Ωt*), and 0, respectively. Under the same driving conditions, the VLPPADA displays the maximum flow rate, and the flow rate of the VLPPSDA is 0. Simultaneously, the pump flow rate of the piezoelectric pump is closely related to the structural parameters of the flow tube.

## 4. Experiments

### 4.1. Experiment Design

**Figure 6:**
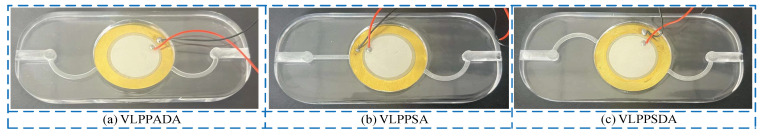
Photographs of these pumps.

Figure 6 shows VLPPs with three different layouts of arc-shaped tubes. Figure 6a presents the VLPPADA, and Figure 6b,c show the VLPPSA and the VLPPSDA, respectively. In order to simplify the production process, facilitate the observation of the internal flow of the flow tube, and avoid liquid leakage, the pump body was manufactured using the SLA (Stereo Lithography Apparatus) technique and made of transparent photosensitive resin with a molding accuracy of ±0.05 mm. The piezoelectric pump can be regarded as an integral unit, and the flow tube was integrated into the pump body. Table 1 shows the structural parameters of the PZT vibrator. It can be seen that the depth of the pump chamber is 1.5 mm, and the diameter is 30 mm. The depth of the arc-shaped tube is 1 mm, and the base circle radius is 12 mm.

**Figure 7:**
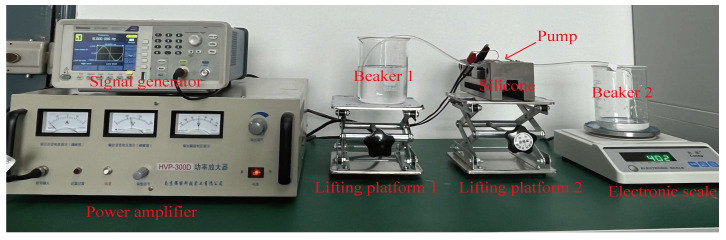
Experimental platform of flow rate test.

Figure 7 shows the flow test platform of the pump, where the working medium is distilled water, the ambient temperature was 25 °C, and the humidity was 60% on the day of this experiment. Each data point was measured 5 times, and the average value was taken. The experimental platform was composed of a power amplifier, signal generator, electronic scale, beaker, and pump. Before this experiment, the silicone tube and pump were filled with water using a syringe, and the internal air was discharged from the pump chamber, flow channel, and silicone tube. During this experiment, beakers with a large diameter were selected, and the lifting platform was adopted to adjust the liquid level height. The liquid level heights in two beakers and the inlet and outlet positions of the piezoelectric pump were kept on the same horizontal line. Under the excitation of the driving signal, the PZT vibrator was deformed, and then the pump could start to pump water. The water was pumped from beaker 1 to beaker 2 through the pump, and the pump flow rate was identified as the increase in value on the electronic scale per unit time.

### 4.2. Results and Discussion

**Figure 8:**
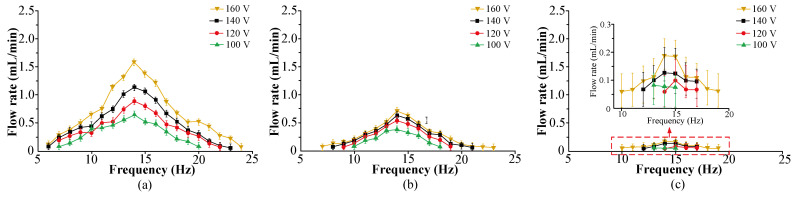
The relationships between the flow rate, the driving voltage, and frequency of the VLPPs: (**a**) VLPPADA, (**b**) VLPPSA, (**c**) VLPPSDA.

Figure 8a shows the relationships between the flow rate, the driving voltage, and frequency of VLPPADA. The experimental results show that the flow rate increases with increasing voltage, and it firstly increases and then decreases with increasing frequency. The maximum flow rate of 1.72 mL/min was achieved when the voltage was 160 V at 14 Hz. This is because at the resonant frequency, the PZT vibrator has the largest vibration amplitude, and the pumping effect of the piezoelectric pump plays a dominated role. At this point, the angular velocity of liquid flow exhibits the fastest value. On the basis of Equation (22), its flow rate reaches the highest value. With the increase in driving voltage, the vibration amplitude of the PZT vibrator and the volume change in the pump chamber will also increase. The pumping effect of the piezoelectric pump becomes more obvious, and the angular velocity of liquid flow becomes larger. On the basis of Equation (22), the flow rate is increased.

Figure 8b shows the relationships between the flow rate, the driving voltage, and frequency of the VLPPSA. The experimental results show that the flow rate increases with increasing voltage, and it firstly increases and then decreases with increasing frequency. The maximum flow rate of 0.77 mL/min was obtained under the voltage of 160 V and frequency of 14 Hz. This is because at the resonant frequency, the PZT vibrator has the largest vibration amplitude, and the pumping effect of the piezoelectric pump plays a dominated role. At this point, the angular velocity of liquid flow reaches a maximum value. On the basis of Equation (27), the corresponding flow rate shows the highest value. With the increase in driving voltage, both the vibration amplitude of the PZT vibrator and the volume change in the pump chamber also increase. The pumping effect of the piezoelectric pump becomes more obvious, and the angular velocity of liquid flow becomes larger. On the basis of Equation (22), the flow rate of piezoelectric pump is increased.

Figure 8c shows the relationships between the flow rate, the driving voltage, and frequency of the VLPPSDA. The experimental results show that VLPPSDA has a small flow rate, and the flow rate reaches 0.16 mL/min when the driving voltage is 160 V at 14 Hz.

**Figure 9:**
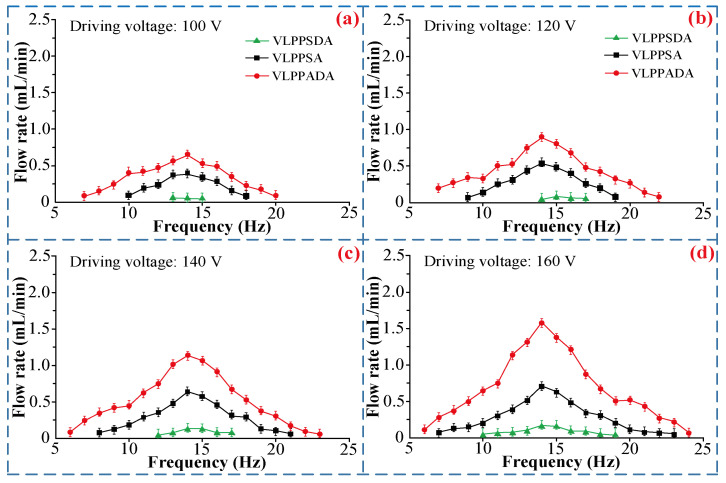
The relationship between the flow rate, the driving voltage, and frequency of VLPPADA, VLPPSA, and VLPPSDA at different driving voltages: (**a**) 100V, (**b**) 120V, (**c**) 140V, (**d**) 160V.

Figure 9 shows the relationships between the flow rate, the driving voltage, and frequency of the VLPPSDA, VLPPSA, and VLPPSDA at different driving voltages. The experimental results show that under the same driving conditions, the flow rate of VLPPSDA exhibits the highest value, while the flow rate of VLPPSDA displays the lowest value, close to zero. It is because within one cycle, the Coriolis forces acting on the two arc-shaped tubes of VLPPADA have the same effect, and their effects are superimposed. The flow rate can be calculated by Equation (22), and its flow rate is the highest. Only one arc-shaped tube has the Coriolis force effect in VLPPSA, and the flow rate can be calculated by Equation (27). Theoretically, its flow rate is half of that of VLPPADA. The Coriolis forces acting on the arc-shaped tubes of VLPPSDA are opposite and cancel each other out. The flow rate can be obtained with Equation (32), and the flow rate should be zero. However, due to the texture directionality of materials such as the arc-shaped tube and pump chamber, the liquid has different flow resistance values during forward and reverse flow, resulting in micro-flow. The above experimental results are consistent with the theoretical derivation, indicating the accuracy of the theoretical analysis.

According to previous work, the flow rate of VLPPADA is the maximum under the same driving conditions [30]. In order to reveal the influence of structural parameters of arc-shaped tubes on flow rate, the different parameters of piezoelectric pumps, shown in Table 2, and the related flow rates were measured.

**Figure 10:**
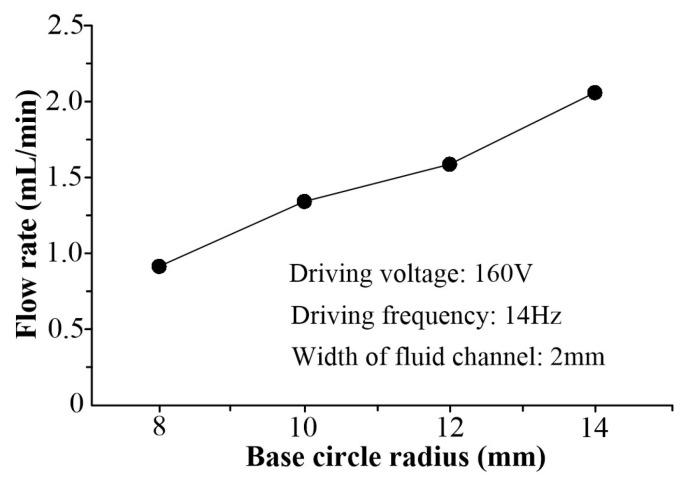
The relationship between the flow rate and the base circle radius.

Figure 10 shows the relationships between the flow rate and the base circle radius when the driving voltage is 160 V at 14 Hz, and the channel width is 2 mm. According to the experimental results, it can be concluded that the flow rate of VLPPADA is enhanced with the increase in the base circle radius under the same driving voltage and frequency, and channel width. This is because as the base circle radius is increased, the Coriolis Effect becomes more significant and the flow rate increases, which is consistent with the result calculated by Equation (22).

**Figure 11:**
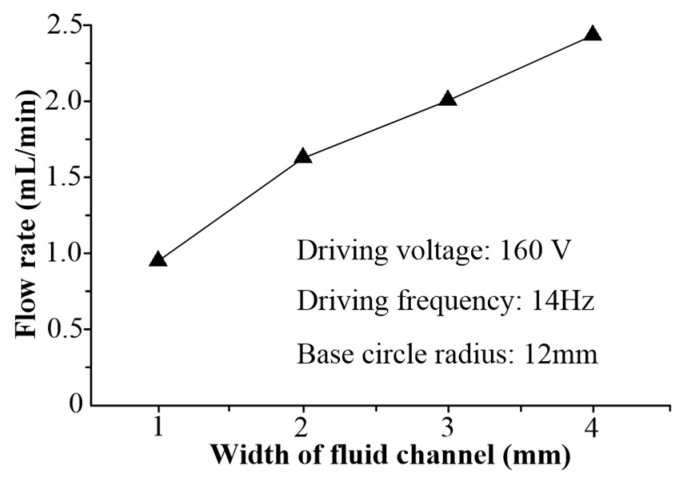
The relationships between the flow rate and the liquid channel width.

Figure 11 shows the relationships between the flow rate and the channel width when the driving voltage is 160 V at 14 Hz, and the base circle radius is 12 mm. According to the experimental results, it can be concluded that the flow rate of VLPPADA is enhanced with the increase in the channel width, under the same driving voltage, driving frequency, and base circle radius. This is because as the width of the channel is increased, the cross-sectional area of the channel is also increased. On the basis of Equation (22), the pump flow rate is also increased.

In summary, the theoretical analysis section assumes the fluid is a viscous ideal fluid and calculates the flow rates of piezoelectric pumps with different flow tube layouts as a trend prediction. To ensure the accuracy of experimental results, the experimental data are averaged from multiple measurements. The experimental results are consistent with the theoretical analysis, verifying the correctness of the theoretical trends through experiments and supporting the experimental findings of this paper with theoretical analysis.

## 5. Conclusions

In this work, the VLPPADA, VLPPSA, and VLPPSDA were designed. The flow characteristics of liquid in arc-shaped tubes were analyzed, and the velocity difference between the clockwise and counterclockwise flow of the liquid were reduced. Eventually, the flow rate equations of the VLPPADA, VLPPSA, and VLPPSDA were established. Three types of circular arc flow tube VLPPs with different layouts were produced using 3D printing technology, and their pump flow rates were measured. The experimental results demonstrate that under equal driving conditions, the flow rate of VLPPSDA reached the highest value, while the flow rate of VLPPSDA was close to zero. Meanwhile, the VLPPADA, with different parameter flow tubes, was produced, and the measured flow rates indicated that the flow rate was directly proportional to the base radius and width of the arc-shaped tube.

## Figures and Tables

**Table 1:** Structural parameters of PZT vibrator.

Title	Diameter of Piezoelectric Ceramics (mm)	Thickness of Piezoelectric Ceramics (mm)	Diameter of Brass Substrate (mm)	Thickness of Brass Substrate (mm)
Size	25.0	0.25	35.0	0.18

**Table 2:** Structural parameters of a VLPPADA.

Pump (Number)	Base Circle Radius (mm)	Width (mm)	Depth (mm)
1	8.0	2.0	1.0
2	10.0	2.0	1.0
3	12.0	2.0	1.0
4	14.0	2.0	1.0
5	12.0	1.0	1.0
6	12.0	3.0	1.0
7	12.0	4.0	1.0

## Data Availability

Data are contained within experiment.

## Abbreviations

The following abbreviations are used in this manuscript:

*I*	quantity in the inertial frame of reference;
*u*	velocity components of the coordinate axis *r*;
*v*	velocity components of the coordinate axis *θ*;
*w*	velocity components of the coordinate axis *z*;
*ρ*	density of the liquid in the flow tube;
P	function of *ρ*;
*a*	base circle radius;
*Ω*	angular velocity of liquid flow;
*v_c_*	velocity of liquid flow clockwise;
*v_a_*	velocity of liquid flow counterclockwise;
*S*	cross-sectional area of the flow channel;
*t*	time of liquid flow;
*v_t(in)_*	velocity of the liquid flows in flow tube B;
*v_t(out)_*	velocity of the liquid flows in tube B.
